# Hotspots, trends, and advice: a 10-year visualization-based analysis of painting therapy from a scientometric perspective

**DOI:** 10.3389/fpsyg.2023.1148391

**Published:** 2023-05-22

**Authors:** Qianrong Liang, Jiarong Ye, Yingyin Lu, Junjie Dong, Heyong Shen, Hongzhong Qiu

**Affiliations:** ^1^School of Finance, Guangdong University of Foreign Studies, Guangdong, China; ^2^Institute of Analytical Psychology, Faculty of Humanities and Social Sciences, City University of Macau, Macau, China; ^3^The Second Clinical College of Guangzhou University of Chinese Medicine (The Second Affiliated Hospital of Guangzhou University of Chinese Medicine), Guangzhou, China; ^4^Department of Social Work, Kunming Children Hospital, Kunming, China; ^5^School of Psychology, South China Normal University, Guangzhou, China; ^6^School of Public Health and Management, Guangzhou University of Chinese Medicine, Guangzhou, China

**Keywords:** painting therapy, bibliometrics, CiteSpace, Web of Science, psychosomatic health

## Abstract

**Purpose:**

Research on painting therapy is available worldwide and painting therapy is widely applied as a psychological therapy in different fields with diverse clients. As an evidence-based psychotherapy, previous studies have revealed that painting therapy has favorable therapeutic effects. However, limited studies on painting therapy used universal data to assemble in-depth evidence to propose a better recommendation on it for the future use. Large-scale retrospective studies that used bibliometric methodology are lacking. Therefore, this study presented a broad view of painting therapy and provided an intensively analytical insight into the structure of knowledge regarding painting therapy employing bibliometric analysis of articles. CiteSpace software was used to evaluate scientific research on painting therapy globally published from January 2011 to July 2022.

**Methods:**

Publications related to painting therapy from 2011 to 2022 were searched using the Web of Science database. This study employed bibliometric techniques to perform co-citation analysis of authors, visualize collaborations between countries/regions as network maps, and analyze keywords and subjects relevant to painting therapy by using CiteSpace software.

**Results:**

In total, 871 articles met the inclusion criteria. We found that the number of painting therapy publications generally trended incrementally. The United States and United Kingdom made the most contributions to painting therapy research and had the greatest impact on the practical application in other countries. *Arts in Psychotherapy* and *Frontiers in Psychology* occupied key publishing positions in this research field. The application groups were mainly children, adolescents, and females, and Western countries paid high attention to painting therapy. The main areas of application of painting therapy were Alzheimer’s disease and other psychosomatic disease fields. Identified research priorities for painting therapy were emotion regulation and mood disorder treatment, personality disorder treatment, personal self-esteem enhancement, and medical humanistic care. Three keywords, “depression,” “women,” and “recovery,” had the strongest citation bursts, which emphasized the research trends.

**Conclusion:**

The general trend for painting therapy research is positive. Our findings provide useful information for researchers on painting therapy to determine new directions in relate to popular issues, collaborators, and research frontiers. Painting therapy holds a promising future, and further studies could explore the clinical implications of this therapy in terms of mechanisms and criteria for assessing efficacy.

## 1. Introduction

In the early 1980s, psychotherapy with various art forms such as painting, music, literature, crafts, and drama became known as “expressive art therapy” at Leiris University, and subsequently developed into an independent field of study. The American Art Therapy Association defines art therapy as an integrative mental health and human services profession that enriches the lives of individuals, families, and communities through active art-making, creative processes, applied psychological theory, and human experience in a psychotherapeutic relationship (Association TAAT, n.d.). As a form of art therapy, painting therapy can capture psychological information that is difficult to obtain from conventional questionnaires, thereby circumventing the limitations of language (Qiu, 2004; Hu et al., 2021). It also helps to break through patients’ psychological resistance and open their hearts, and allows them to share their feelings, views, and emotions with others. Painting therapy has psychological therapeutic effects such as cultivating emotional resilience, resolving distress, and fostering self-confidence (Qiu, 2004; Puetz et al., 2013; Hu et al., 2021). It is widely applied as a psychological therapy in different fields with diverse clients, including individuals with emotional problems (Qiu et al., 2017), children or adolescences with special needs (Klop, 2017; Durrani, 2019), and patients with mental illnesses (MacIntosh, 2017). In addition, painting therapy can address the psychological needs of patients with cancer (Czamanski-Cohen et al., 2019; Sw et al., 2019; Thyme et al., 2022), Parkinson’s disease (Schofield, 2019), diabetes (Zamanifard et al., 2022), functional gastrointestinal diseases (Kai et al., 2022), and other physical illnesses. Although research on this evidence-based psychotherapy is available worldwide and the amount of its publications have emerged, limited studies on painting therapy have used universal data. Large-scale retrospective studies that used bibliometric techniques are also lacking. It is significantly essential to summarize the current studies on painting therapy by bibliometric approach.

Bibliometrics has become an influential method for in-depth quantitative analysis and is a scientific tool that can be used to investigate hotspots and reveal trends in a certain research area (Durieux and Gevenois, 2010; Huertas González-Serrano et al., 2020). This approach refers to the study of the distribution, quantitative relationships, and change patterns of a field of study by taking the measurement characteristics of the literature as the object of study and then exploring the structure, characteristics, and patterns in a subject area through the filtering and processing of massive amounts of information. It can also reveal the evolution of a journal (Chen et al., 2022; Envelope et al., 2022). It applies mathematics, statistics and computer science to all sorts of documentary data with the competence to identify trends in the development of various disciplines and dig out the potential value of knowledge (Yu et al., 2018). It has been widely used in the field of engineering and technology (Yu et al., 2018, 2020; Lin et al., 2021; Chen et al., 2022; Envelope et al., 2022), humanities and social sciences (Tal and Gordon, 2018), medicine (Chen et al., 2022; He et al., 2022), economics and management (Zhong and Lin, 2022; Zhang et al., 2023).

CiteSpace, developed by Chen (2004), is a Java application software for data analysis and visualization based on the theory of co-citation analysis. It is a unique and leading application for information visualization analysis using three major concepts: burst detection, betweenness centrality, and heterogeneous networks. These three concepts can recognize the character of research frontiers, mark keywords, and identify emerging trends and sudden changes over time, respectively (Chen and CiteSpace, 2006). CiteSpace evaluates co-citations, co-authors, and co-occurrence keywords and analyzes the data using procedural steps: time slicing, thresholding, modeling, pruning, merging, and mapping (Chen, 2004). Analysis using CiteSpace is beneficial for users to obtain key information regarding the research field under study and recognize the trends in a certain area.

Due to the lack of bibliometric analysis of painting therapy, quantitative results on the use, collaboration, exchange and research of painting therapy internationally, as well as on the future direction of research development are unclear. The Citespace-based bibliometric analysis may address this issue, providing a comprehensive quantitative overview, display a broad panorama and visual analysis on the current state and the hotspot of painting therapy. Therefore, this study presented a broad view of painting therapy and provided an intensively analytical insight into the structure of knowledge regarding painting therapy employing bibliometric analysis of articles.

This paper used bibliometric analysis and social network analysis to analyse the publications on the painting therapy during 2011–2022. To enhance understanding of the field in particular, the main contributions of this paper are as follows: (1) Discussions on the published articles were conducted at the national/regional, institutional and author levels, followed by a cluster analysis based on the research literature on painting therapy in order to understand the topical issues in this field. (2) Social network analysis was used to quantify the extent of national and institutional collaboration quantitatively. (3) Analysis of the content of key literature qualitatively provides deep insight into issues of close interest to the authors, as well as future research trends. (4) In-depth discussions were held from the perspective of current burning issues, future trends and challenges, and limitations.

## 2. Materials and methods

### 2.1. Search strategy

The data used in this study were obtained from Web of Science (WoS), a database developed by Thomson Scientific and has been used in tens of thousands of academic studies. It is one of the most trustworthy international citation datasets, covering a large amount of literature published from 1900 to the present and providing journals with high quality and information about literature worldwide in details (Chen et al., 2022). It is not only applied as a research tool to support a variety of scientific tasks in various domains of knowledge, but also as a statistic set for large-scale data-intensive scientific research.

Art therapy was chosen as the search term since painting therapy belongs to it. However, painting therapy may be expressed in a variety of ways. It can also be named after different forms of painting. In order to collect more comprehensive literature and cover a wider range of painting therapy modalities to analyze the current state of painting therapy, various forms of painting were included as search terms in this article. Combination Antiretroviral Therapy (cART) and Assisted Reproductive Technology (ART), as the abbreviations are the same as ART, have been added to the exclusion terms to exclude literature that is not relevant to painting therapy. In addition, painting therapy is not belonged to art education, and articles on art education were also excluded. Therefore, we performed data acquisition on July 26, 2022 using the following search terms: title = (((((TS = (Art Therapy OR art therapy OR arts therapies)) OR (((TI = (Art OR arts))OR (TI = (artists*))))) OR ((((((((((((((TI = (draw* OR paint*))) OR (TI = (sketch))) OR (TI = (coloring*))) OR (TI = (doodle))) OR (TI = (doodling))) OR (TI = (collage*))) OR (TI = (craft*))))) OR (TI = (expressive*))) OR (TI = (Tracing))) OR (TI = (still life))) OR (TI = (Mandala)))) NOT (AB = (cART OR combination antiretroviral therapy OR art education OR HIV OR Assisted Reproductive Technology)) NOT (TS = (cART OR combination antiretroviral therapy OR art education OR HIV OR Assisted Reproductive Technology))) NOT ALL = (cART OR combination antiretroviral therapy OR art education OR HIV OR Assisted Reproductive Technology)) and time span = 2011–2022.

### 2.2. Inclusion criteria

The title field was painting therapy, and “articles” was the only document type selected in the advanced search. Other document types, such as editorial material, letters, and book reviews, were excluded. 1,297 articles were returned in this advanced search process.

Irrelevant documents were removed by manually filtering each article when downloading the data. These irrelevant documents mainly included documents with the search terms in the subject but that were not about painting therapy (e.g., combination antiretroviral therapy in the medical field) and articles on non-painting therapy such as music therapy, dance therapy, art exhibitions, art professions, art taste, and other unrelated articles. Removal of duplicates was performed using CiteSpace. Finally, 871 valid documents were identified as the data source for this study.

### 2.3. Data extraction

The authors extracted publications and used Microsoft Excel 2016 and EndNote to analyze the publications obtained from the WoS database. In addition, basic information for each publication were downloaded and recorded as bibliometric indicators, including citation frequency, authors’ country or region, and journal. The Hirsch index (H-index) is used to measure the citation frequency of research in academic journals or researchers (Wang et al., 2019). The IF and the H-index were used to evaluate journal quality and scientific research influence. The IF is a measure of the number of citations to articles published in a journal over a 2 year period. It is a widely recognized and widely used evaluation index that reflects the overall quality of the journal, but it fail to measure the impact of a specific article or research scholar. The H-index, on the other hand, can reflect the quality of most articles published in the field of painting therapy rather than identifying individual papers with more citations, which is more accurate and targeted than the IF in an analysis of a specific field. The H-index is a delicate balance between the number of papers and their citation rate, thus reducing the ‘over-rating’ of smaller journals. In fact, the H-index and the IF are like a complementary relationship, and the two together make for more reliable journal evaluations. In scientific research literature, keywords often reflect the main research content of the literature. Therefore, clustering analysis of keywords can aid in understanding the development lineage, main research contents, and research frontier hotspots of a particular research field. What’s more, the keyword co-occurrence network can reflect current research hotspots as well as hotspots that were previously generated in the field. Centrality is a key indicator for analyzing the importance of keywords. The greater the centrality, the greater the importance and influence of the node in the study. The combination of node size, centrality and frequency of keyword occurrence reveals the focus and hotspots in the research field.

### 2.4. Analysis methods

The goal of bibliometrics can be depicted as the performance of research that contributes to the development of the field of knowledge by inferring and interpreting relevant analyses (Castanha and Grácio, 2014; Merigó et al., 2019; Mulet-Forteza et al., 2021). As a bibliometric software, CiteSpace produces information to visualize data better. This software was used to visualize scientific maps for publications on painting therapy from 2011 to 2022 with years per slice (slice length = 1), author co-citation networks, inter-country and inter-regional collaboration networks and network maps, co-cited journals, and co-occurring keywords for top painting therapy research (timeline view). Co-citations occur when two articles receive citations from the same third study (Henry, 1973; Merigó et al., 2019). Every option in the terminology source was selected, and a single node type was chosen at a time based on particular conditions. The “top 25 levels” were used as thresholds for the most frequently cited or referenced articles in the corresponding time slice. Nodes and links were essential elements to develop a visualization knowledge figure. A node represents an element (e.g., an author, a keyword, a region, or a country) with the node’s size proportional to the occurrence or citation frequency, and the node’s color indicates the year. Furthermore, each node is represented by a series of concentric circles or “tree rings” on a time slice. The concentric circles’ size indicates the publications’ number, and different colored circles from the inside to the outside of a node indicate the years (2011–2022). Links between two nodes represent collaboration, co-occurrence, or co-citation relationships. The green ring represents the mediated centrality of the literature (Chen et al., 2009), whereas the red indicates the time slice where sudden rise in citations or detectable citation bursts happened (Kleinberg, 2002).

Scientific maps are usually featured as a group of points and lines to indicate collaboration among publications (Chen and CiteSpace, 2006). A point is used to signify an author, country/region, journal, or keyword, whereas a line represents a link between points, with wider lines indicating stronger links (Zheng and Wang, 2019). Moreover, a scientific map includes nodes that represent the frequency of citations for certain topics. Burst nodes in a central red circle form indicate increasing co-occurrence or number of citations over time. The purple nodes represent centrality and suggest essential knowledge is shown by the data (Chen and CiteSpace, 2006; Chen et al., 2012; Zheng and Wang, 2019). Occurrence bursts in the map represent the topics’ frequency, whereas citation bursts indicate the references’ frequency (Chen and CiteSpace, 2006). Citation bursts for keywords and references can be used to explore trends and indicate whether particular authors were gaining considerable attention in the field (Chen and CiteSpace, 2006). These maps build understanding of emerging trends and help to track hot topics through burst detection analysis (Liang et al., 2017; Miao et al., 2017).

## 3. Results

### 3.1. Publication outputs and time trends

**Figure 1:**
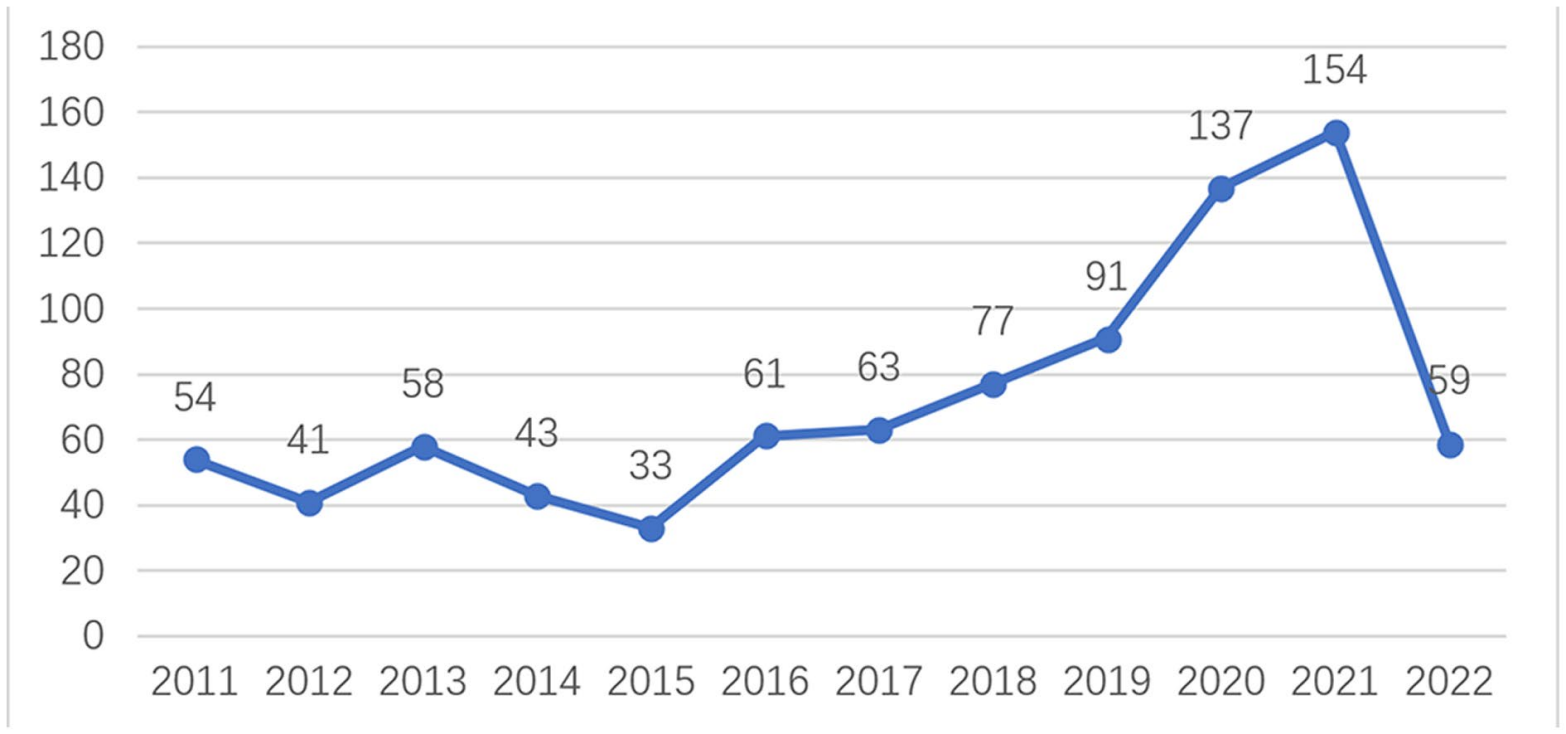
Time sequence of relevant articles on painting therapy published from 2011 to 2022 in Web of Science.

As can be seen in Figure 1, the number of articles on painting therapy was relatively stable between 2011 and 2017, with about 40–60 publications per year, and then steadily grew from year to year. This showed that increasing attention has been paid to painting therapy since 2017. There was a more rapid increase in the number of articles between 2019 and 2020, and in 2020, the number of articles on painting therapy exceeded 100 for the first time. The average annual number of publications in the last 2 years has been in excess of 130. Overall, the general trend in the number of articles published on painting therapy was incremental. In 2022, however, as of July 2022, only 59 papers were published in this approximately six-month period, which is less than the number of articles in 2020 and 2021. This needs to be followed up further.

### 3.2. Distribution by country or region

Studies published from 2011 to 2022 in the WoS database were selected and analyzed with a time slice of 1 year. This included 871 manuscripts on painting therapy, which were published in 61 countries and regions. All articles are written in English. CiteSpace was used to analyze the distribution of these articles in terms of which countries or regions they were published in. The node type was selected as “country,” g-index (*k* = 25) as the threshold, and slice = 1. The top 10 countries or regions are highlighted in Table 1. The average article citations, total citations on WoS, and H-index for these top 10 countries or regions are also presented. The H-index is used to measure the influence of authors’ scientific achievements. The United States had the most published manuscripts (222 studies), together with the most citations on WoS (2,157 citations), and the highest H-index value (*H* = 38). Its average number of article citations (9.72 citations) was ranked second. The United Kingdom had the second largest number of publications (102 studies) and a total of 999 citations on WoS. It had the highest average number of citations (9.79 citations). These findings indicated that the United States and the United Kingdom, as the countries with the earliest application of painting therapy, made the most contribution to the field of painting therapy research and had the greatest impact on the practical application of this therapy in other countries. This laid a research foundation for painting therapy development.

**Table 1:** Top ten countries or regions that published articles related to painting therapy.

Country	Publications	Frequency	H_index	Total citations	Citations per paper
USA	222	0.255	38	2,157	9.72
United Kingdom	102	0.117	32	999	9.79
Israel	87	0.1	13	437	5.02
Germany	60	0.069	21	476	7.93
Canada	56	0.064	25	456	8.14
China	47	0.054	15	213	4.53
Australia	39	0.045	17	153	3.92
Korea	33	0.038	12	255	7.73
Netherlands	26	0.03	15	150	5.77
Italy	25	0.029	9	123	4.92

**Figure 2:**
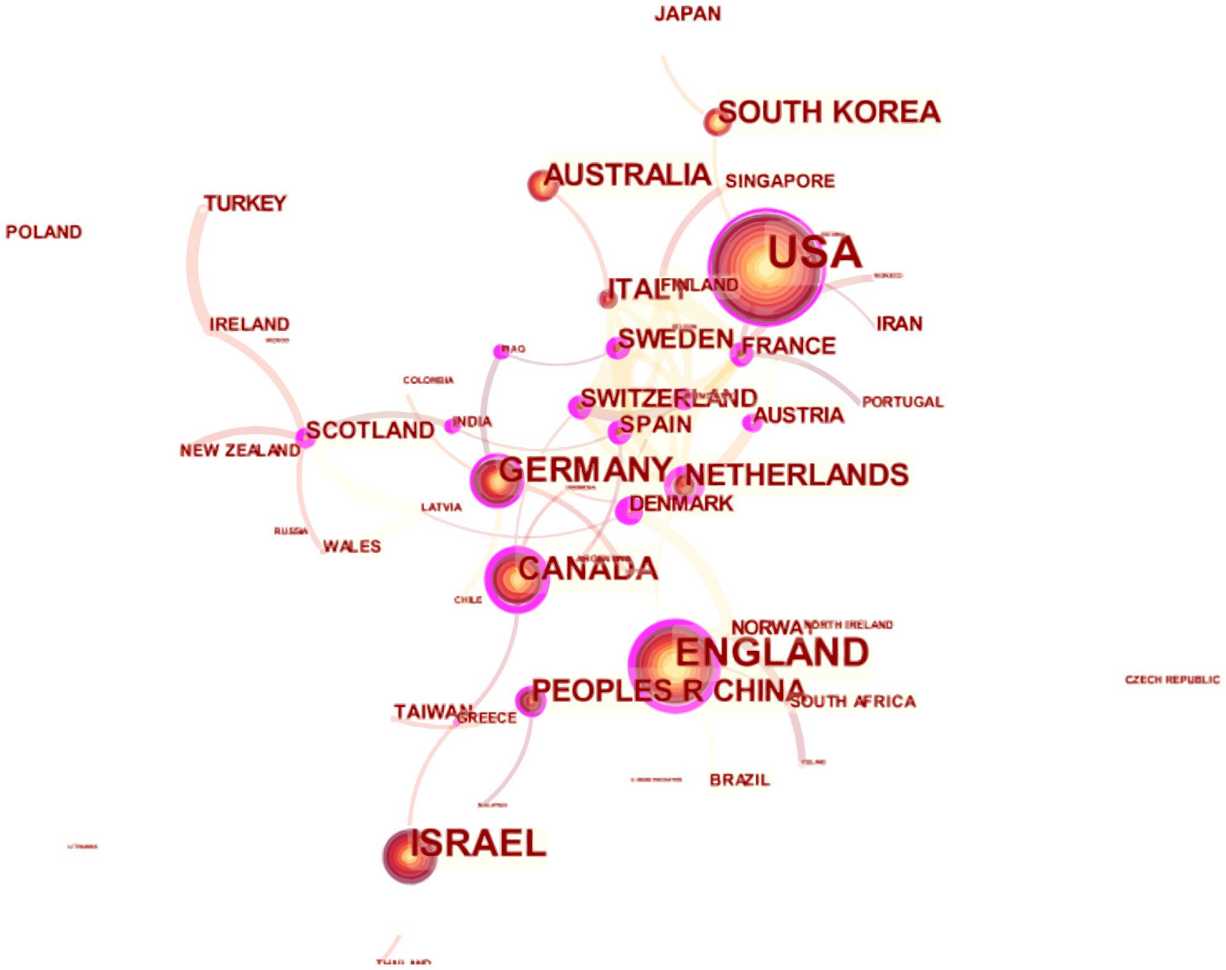
The collaboration networks among countries or regions. In this map, the circle represents a country or a region, whose size reflects the amount of publications in a certain country. Thicker lines between nodes indicate more cooperative publications. The shorter the distance between two nodes, the stronger cooperation between the two countries or regions.

Figure 2 displays the collaboration networks among countries or regions. In total, 61 nodes and 124 links were contained in the collaboration networks. The size of the circle represents the amount of publications in a certain country. The largest node was located in the United States with the maximum publication count on painting therapy. In addition, a lighter color indicates a study was published earlier. Thicker lines between nodes reflect more cooperative publications. The shorter the distance between two nodes, the stronger cooperation between the two countries or regions. We observed that in the early stages of research on painting therapy, there was cooperation between European countries, with other countries around the world engaging in a greater level of cooperation in research as the field deepened and developed. The darkest purple circle was shown for Spain (*H* = 0.5), meaning that Spain had the greatest amount of collaborations with other countries or regions in this research field.

### 3.3. Distribution by journals

**Figure 3:**
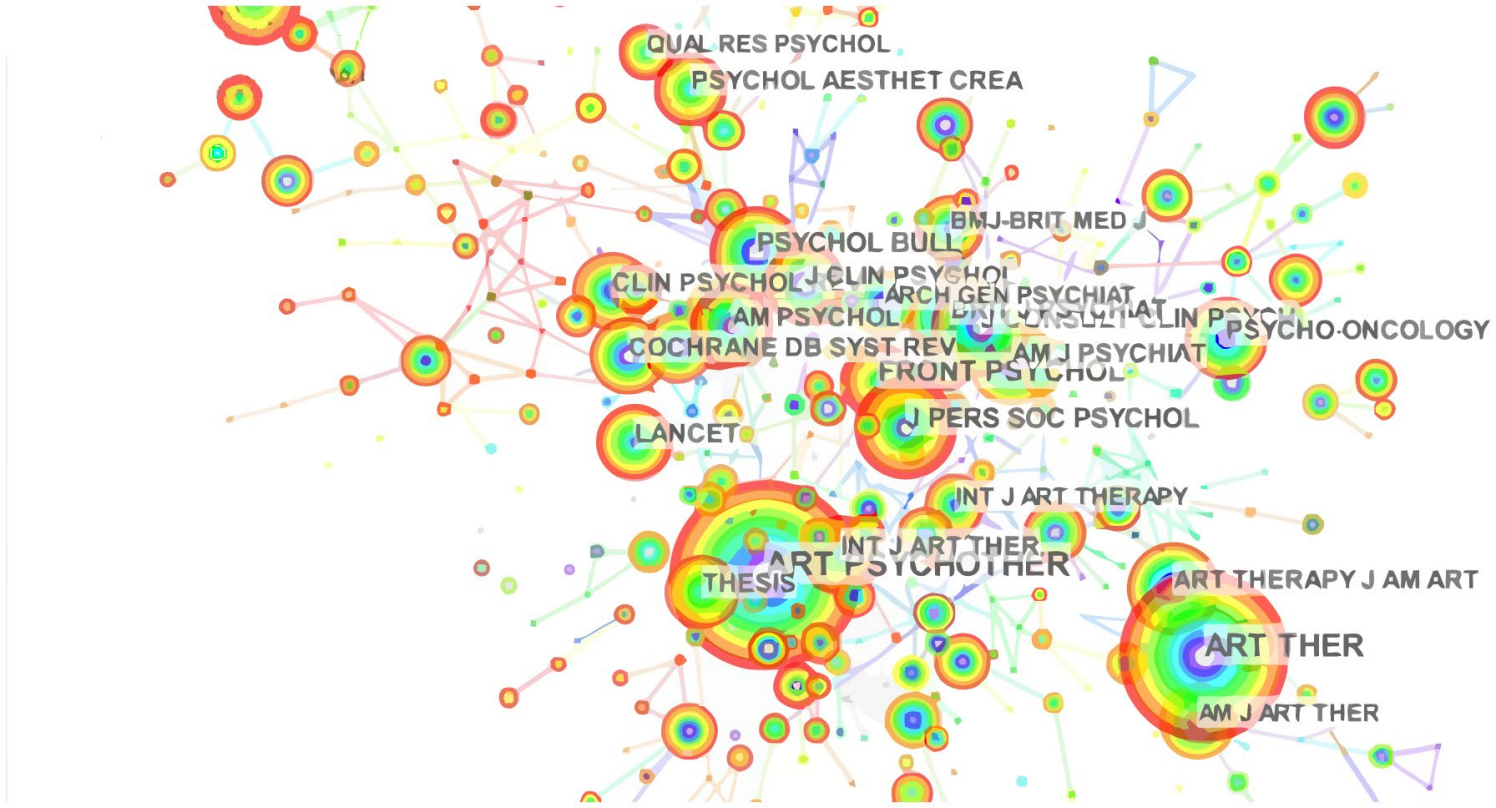
The map of the co-citation journals. In this map, the node represents a journal. The larger the node, the more publications in that journal. The link between two nodes represents the frequency of co-citation, and a thicker purple circle suggests greater impact on the field of painting therapy.

Table 2 lists the top 10 journals publishing manuscripts related to painting therapy, along with the journals’ impact factors (IFs) and H-index. It shows that articles were mostly published in *Arts in Psychotherapy*, *Frontiers in Psychology*, *Psychology of Aesthetics Creativity and the Arts*, *Disability and Rehabilitation*, and *Dementia-International Journal of Social Research*. The IFs of these journals varied between 1.037 and 4.232 (average IF: 2.472). Seven of these top 10 journals had an IF > 2, of which *Frontiers in Psychology* had the highest IF for 2022 (4.232). In addition, *Arts in Psychotherapy* (2022 IF: 1.847) published 222 articles on painting therapy in the past decade, and had the highest H-index (*H* = 20), followed by *Frontiers in Psychology* (83 publications, *H* = 8), and *Psychology of Aesthetics Creativity and the Arts* (13 publications, 2022 IF: 2.325, *H* = 5). Figure 3 presents the map of the co-citation journals, which included 534 nodes and 1,862 links. A journal is represented by a node in this co-citation map. The larger the node, the more publications in that journal. The link between two nodes represents the frequency of co-citation, and a thicker purple circle suggests greater impact on the field of painting therapy. A high co-citation count identifies journals with the greatest academic impact that occupy the key position in the research field. *Arts in Psychotherapy* had the greatest number of co-citation count at 698, followed by *Frontiers in Psychology* (158 citations), and *PLOS One* (140 citations). Thus, based on the publications and co-citation counts analysis, *Arts in Psychotherapy* and *Frontiers in Psychology* held key positions in the painting therapy field.

**Table 2:** Top 10 journals publishing manuscripts related to painting therapy.

Journal	IF (2022)	H_index	Publications
Arts in Psychotherapy	1.847	20	222
Frontiers in Psychology	4.232	8	83
Psychology of Aesthetics Creativity and the Arts	2.325	5	13
Disability and Rehabilitation	2.439	8	10
Dementia-International Journal of Social Research and Practice	2.624	5	8
British Journal of Guidance and Counselling	1.125	3	8
Australian and New Zealand Journal of Family Therapy	1.037	2	8
European Journal of Oncology Nursing	2.588	4	7
BMJ Open	3.006	4	6
Family Process	3.497	4	6

### 3.4. Distribution by authors

**Figure 4:**
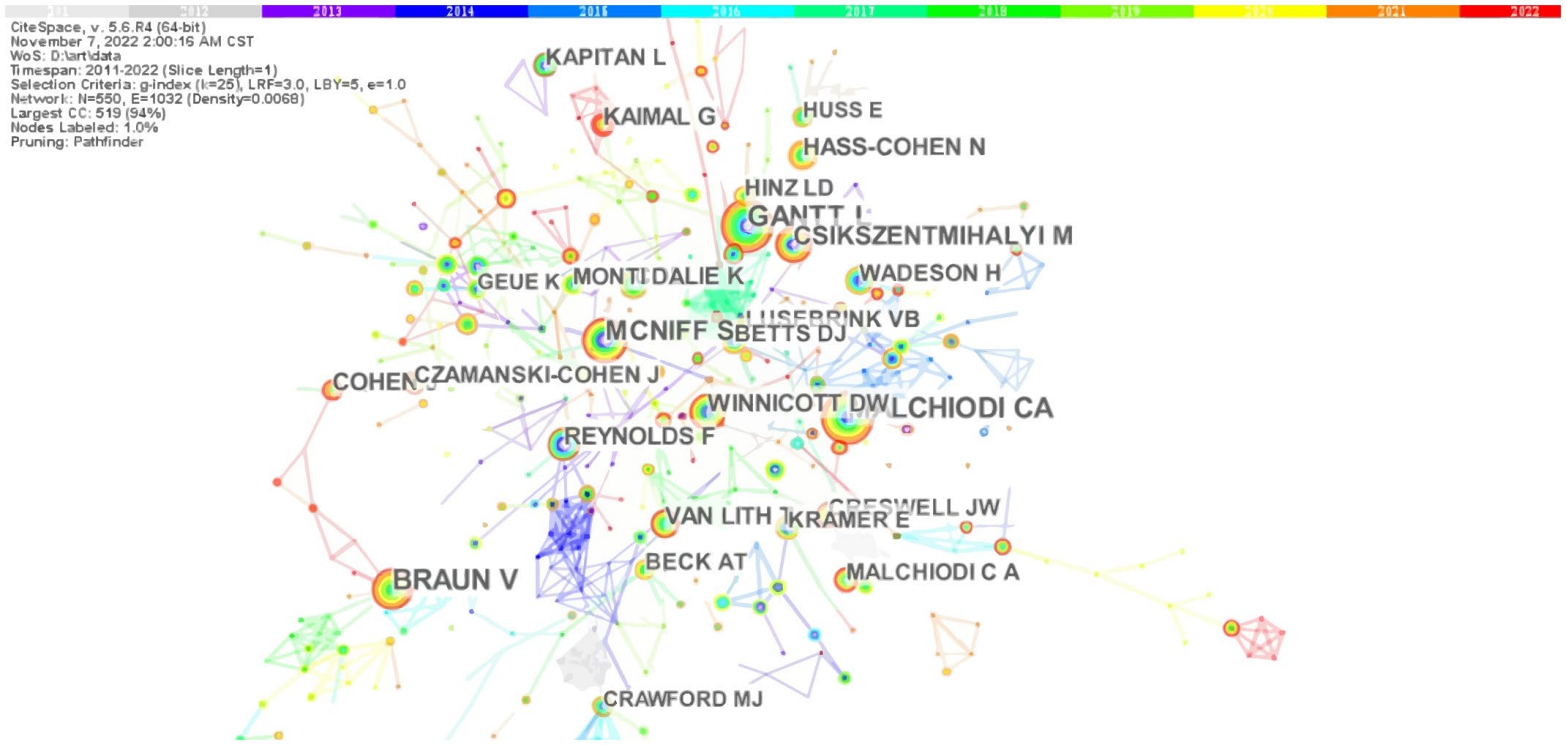
The map of the co-citation authors. In this map, the node represents a journal. The larger the node, the more publications in that journal. The link between two nodes represents the frequency of co-citation, and a thicker purple circle suggests greater impact on the field of painting therapy.

A total of 711 authors had contributed to the research in the field of painting therapy. Table 3 presents the top 10 authors publishing manuscripts studies related to painting therapy according to the output amount and citations by others. However, the overall publication counts were not large. Author Levwiesel R published the most manuscripts (10 publications), followed by Snir S (nine publications) and Kaimal G (nine publications). For co-citation counts, Braun V (65 citations) and Malchiodi CA had the most co-citations, followed by Gantt L (61 citations), McNiff S (51 citations), and Csikszentmihalyi M (38 citations). Figure 4 shows the co-citation network. These nodes represent the number of articles published by each author: a larger node indicates more publications, and a shorter distance between two nodes reflects more cooperation between authors. The color of the circle highlighted the author of the same cluster. Blue circles mean earlier outputs, whereas yellow circles represent more recent publications. The large-sized node that represented authors Braun V, Malchiodi CA, and Gantt L, indicated that these authors were the most co-cited authors, which can be regarded a remarkable progress in the field of painting therapy.

**Table 3:** The top 10 authors publishing manuscripts studies related to painting therapy according to the output amount and citations by others.

Number	Author	Co-citation counts	Author	Publications
1	Braun V	65	RACHEL LEVWIESEL	10
2	Malchiodi CA	65	SHARON SNIR	9
3	Gantt L	61	GIRIJA KAIMAL	9
4	McNiff S	51	LIMOR GOLDNER	8
5	Csikszentmihalyi M	38	DAFNA REGEV	8
6	WINNICOTT DW	36	SUZANNE HAEYEN	8
7	Hass-Cohen N	35	EPHRAT HUSS	7
8	Van Lith T	35	VICKY KARKOU	6
9	Reynolds F	34	HOD ORKIBI	5
10	Collie K	33	MICHAL BAT OR	4

### 3.5. Analysis of keywords

The keywords analysis showed the research hotspots and provided a method to identify further research topics. Table 4 displays the top 30 keywords that had the most frequent use (e.g., “Alzheimer’s disease/dementia”), the method or function of painting therapy (e.g., “psychotherapy,” “drawing,” and “experience”), and the applied groups (e.g., “adolescent,” “women,” and “children”). As the mediated centrality can also reflect research hotspots to some extent, this study comprehensively considered the centrality and frequency of keywords as the index to judge research hotspots. The keyword “Alzheimer’s disease/dementia” had the highest centrality (0.17/0.13).

**Table 4:** The top 30 keywords with the most frequency and centrality in painting therapy field.

Number	Publications	Centrality	Keywords
1	92	0.07	Depression
2	82	0.09	Children
3	72	0.05	Intervention
4	62	0.13	Mental health
5	56	0.03	Quality of life
6	55	0.06	Anxiety
7	52	0.11	Psychotherapy
8	50	0.15	Adolescent
9	39	0.03	Health
10	39	0.03	Symptom
11	38	0.01	Trauma
12	37	0.10	Women
13	34	0.00	People
14	34	0.03	Model
15	34	0.10	Drawing
16	34	0.10	Experience
17	34	0.13	Dementia
18	33	0.10	Life
19	33	0.13	Cancer
20	32	0.07	Scale
21	31	0.05	Creativity
22	30	0.03	Program
23	30	0.05	Disorder
24	30	0.10	Breast cancer
25	29	0.03	Impact
26	28	0.02	Care
27	27	0.03	Mindfulness
28	25	0.11	Schizophrenia
29	23	0.03	Emotion
30	23	0.17	Alzheimers disease

**Figure 5:**
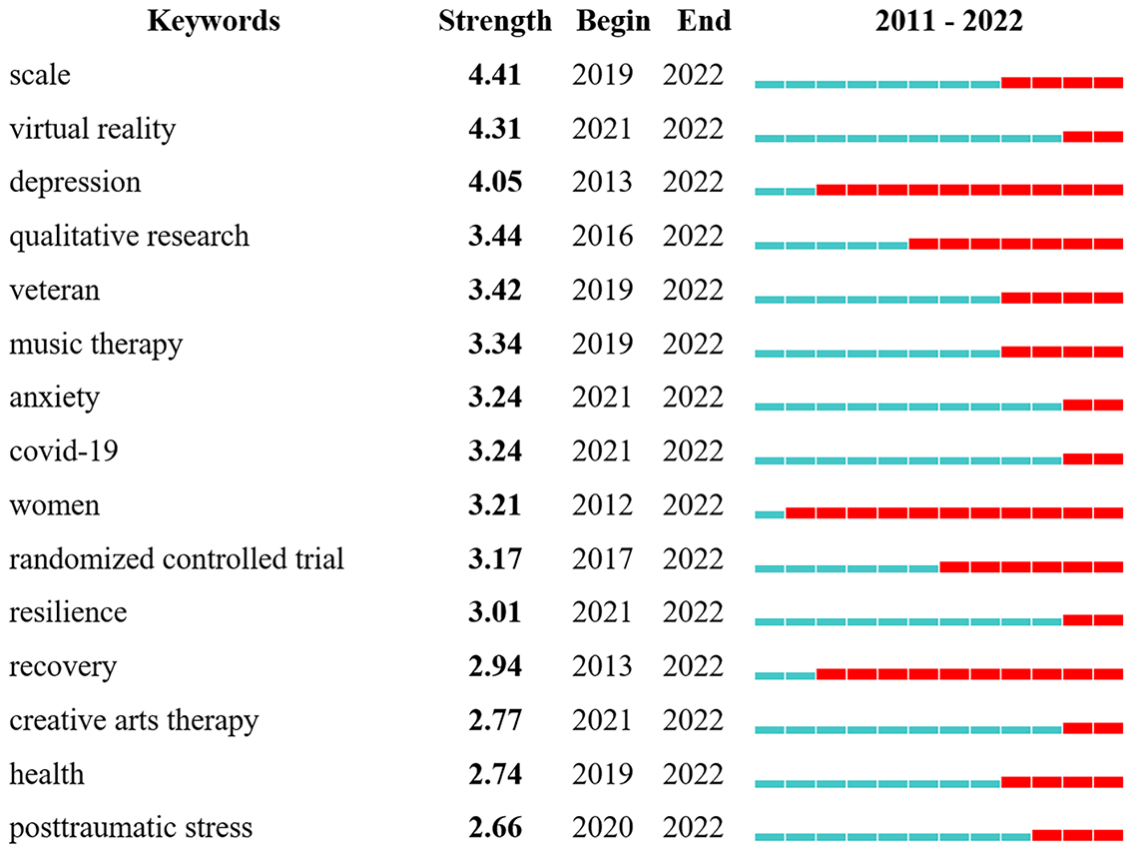
The top 15 keywords with the strongest citation bursts. The blue measures reflect infrequent citation of keywords while the red measures represent frequent citation of keywords.

Figure 5 presents the top 15 keywords with the strongest citation bursts. “Depression” was one of the strongest keywords bursts (4.05) and highlighted the emerging trend of painting therapy studies in the recent decade. The words “women” and “recovery” were similar to “depression,” with bursts of 3.21 and 2.94, respectively. Keywords that received major attention in 2022 and formed the focus of current painting research were “virtual reality” (4.31), “anxiety” (3.24), “COVID-19” (3.24), “resilience” (3.01), and “creative arts therapy” (2.77).

**Figure 6:**
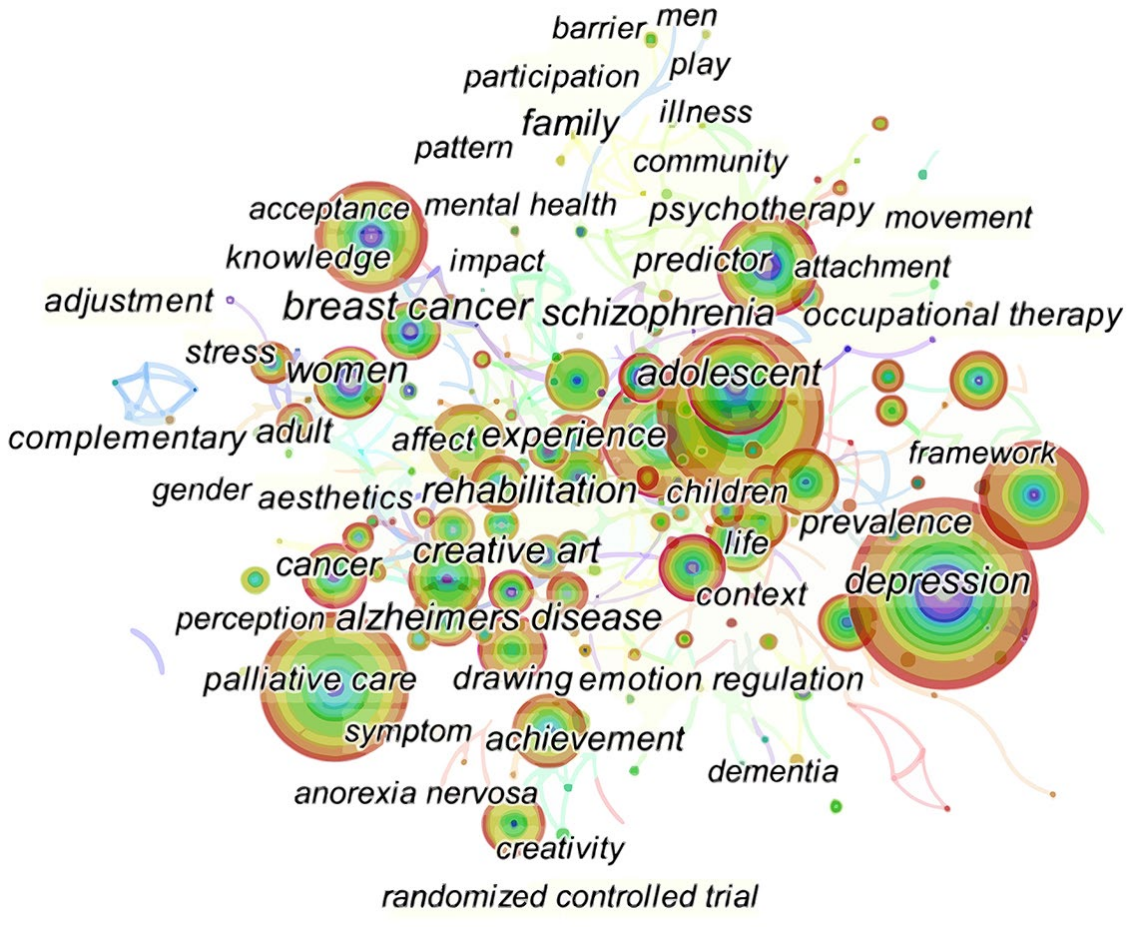
The keyword co-occurrence network map. In this map, the node represents a keyword and the link represents the co-occurrence frequency. The larger the node, the more publications of a certain keyword. A thicker purple circle suggests greater impact in this field. Colored lines represented by time zone that appeared between items: the red can be considered as the newest, and the light grey the oldest.

Co-occurring keywords reflected research hotspots in painting therapy field. With “Keywords” as the node type, the g-index (*k* = 25) as the threshold, slice = 1, pathfinder as the network trimming method, and other parameters as default values, 405 nodes and 1,377 inter-node links were obtained. After the keyword “art therapy” and the words belonging to that phrase (“art” and “therapy”) were hidden, the keyword co-occurrence network map was obtained as shown in Figure 6.

**Figure 7:**
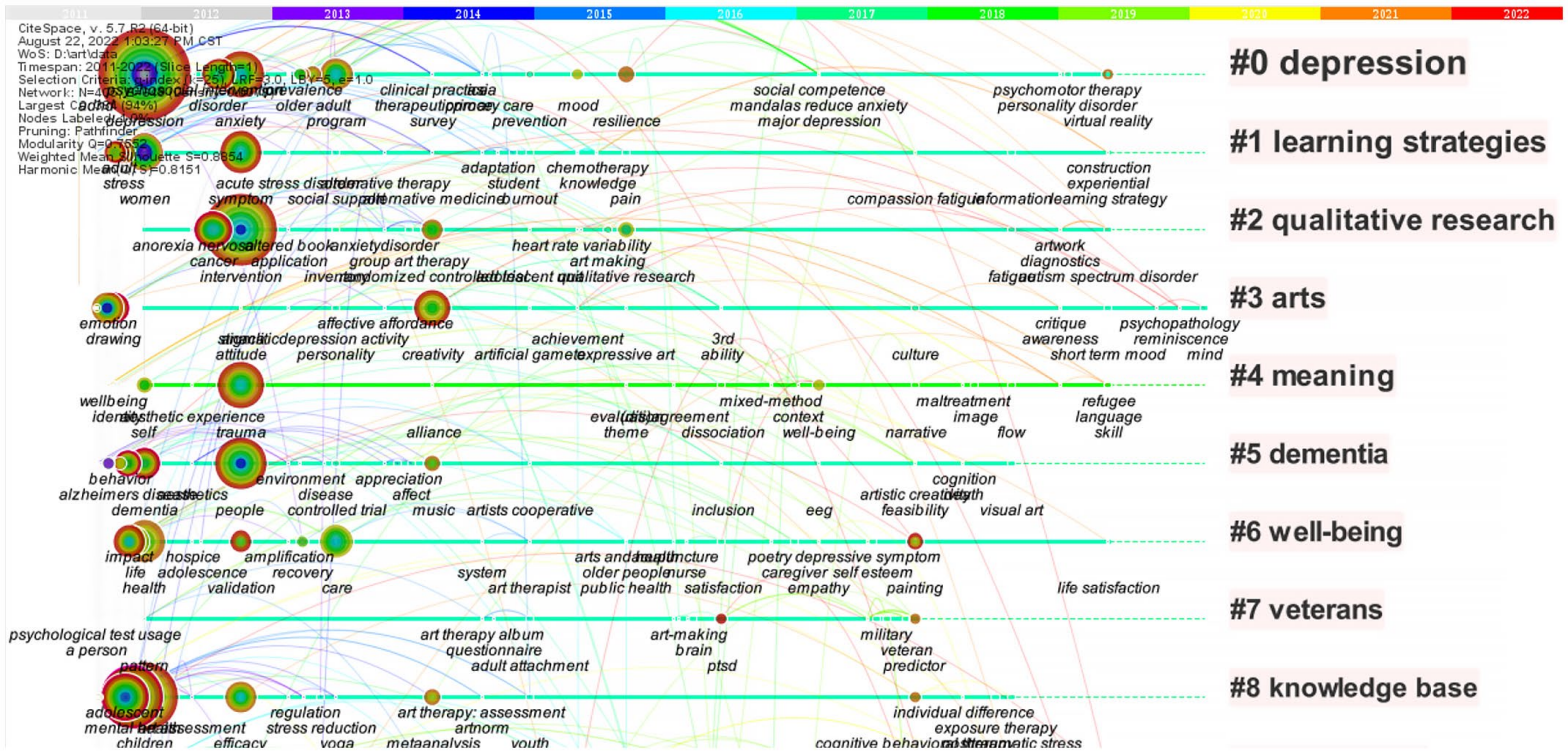
Recurring research on painting therapy after Figure 6 data are sorted into chronological order. In this map, nodes corresponded to keywords, and the size of the nodes indicated the frequency of co-occurring keywords. The nodes on the same line belong to the same cluster.

In this network map, nodes corresponded to keywords, and the size of the nodes indicated the frequency of co-occurring keywords. Colored lines represented by time zone that appeared between items: the red can be considered as the newest, and the light grey the oldest. The top 30 most cited or co-occurring keywords were selected from each slice. The highest frequency was for “depression” at 94, followed by “children,” “intervention,” “mental health,” and “quality of life.” Most of the nodes marked with purple circles represented good betweenness centrality, and suggested that these items were essential. In other words, these nodes with the strongest bursts reflected emerging tendency in painting therapy field. Figure 6 was sorted by the chronological order to obtain Figure 7, which displays the chronological view of the painting research field. The historical progression of clusters reflected the development of research on painting therapy over a specific period of time and the thematic concentration of each cluster.

“Depression” formed the largest cluster (#0), followed by “learning strategies” (#1) and “qualitative research” (#2). “Depression,” “arts” (#3) and “well-being” (#6) continued to be the popular topics in last few years, indicating that they were classical topics for painting therapy. In addition, Clusters 0 and 6 had more keywords appearing in 2019–2020 than other clusters of classic topics, with “personality disorder” in Cluster 0 and “self-esteem” in Cluster 6. The horizontal line in “compassion” (#9) was lighter in color and had recent keywords such as “medical humanity” and “emotion regulation.” The recent keywords in the above clusters suggested that researchers recently investigated areas related to these keywords, meaning these clusters can be considered the frontiers of painting therapy research. Therefore, it can be predicted that painting therapy for emotion regulation and mood disorder treatment, personality disorder treatment, personal self-esteem enhancement, and medical humanity care will be the major focus of further research in the painting therapy field internationally.

### 3.6. Analysis of subjects

**Figure 8:**
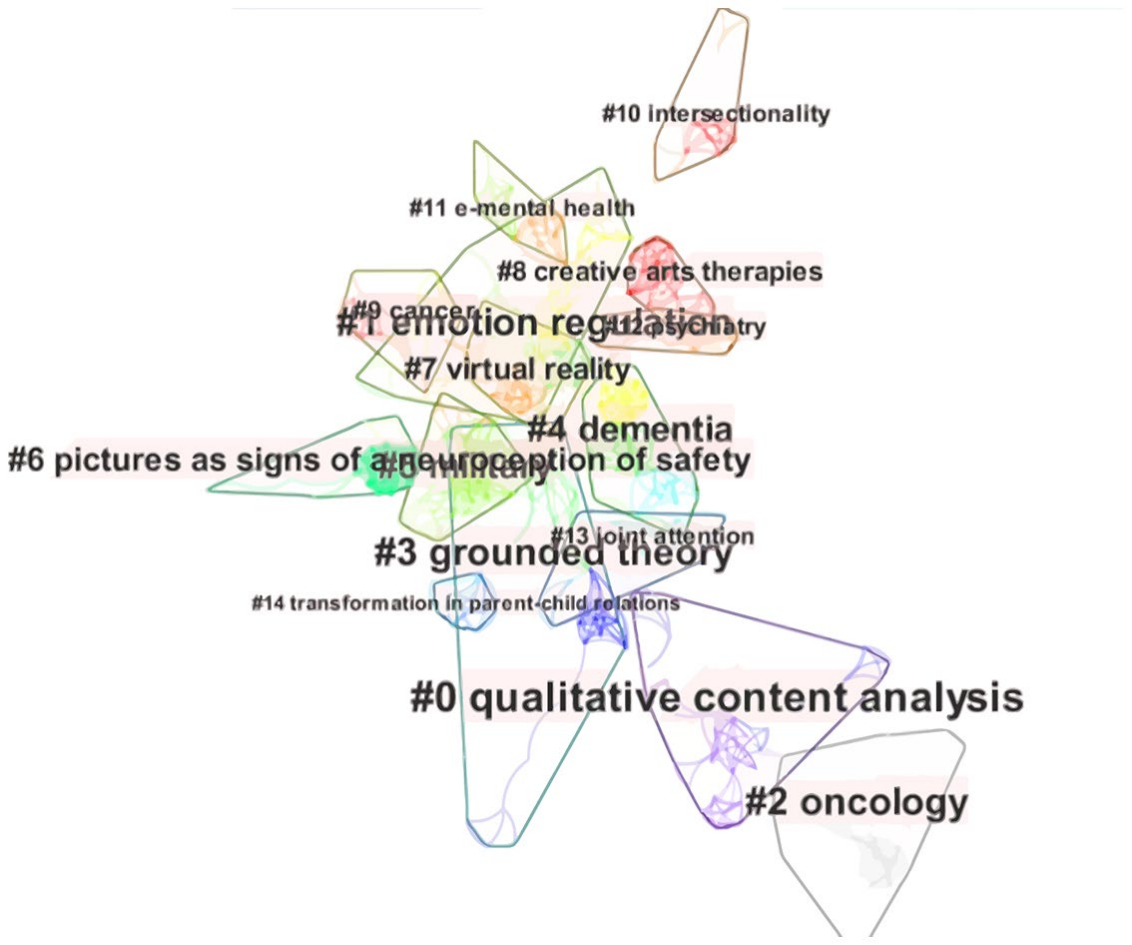
The subjects clustered using the log-likelihood ratio algorithm. In this map, each cluster contained a set of closely related keywords which formed a certain subject. The smaller the number of the cluster, the more keywords it contained, and the larger the corresponding area. The corresponding time was distinguished by the cluster’s color, with cooler colors signifying earlier years and warmer colors indicating more recent ones.

A set of closely related keywords formed a certain subject. The subjects were clustered using the log-likelihood ratio algorithm and the results were labeled according to the importance level to obtain the subject clustering, as shown in Figure 8. Two indicators, modularity Q and the contour value S, were used to evaluate clusters. *Q* values >0.3 suggest that the network is clear and essential, *S* values >0.5 mean that the clustering results are ideal and > 0.7 imply the results are reliable (Jin et al., 2022). In this analysis, the modularity *Q* was 0.8306 and *S* was 0.9293, reflecting that the results were reliable and meaningful. The smaller the number of the cluster, the more keywords it contained, and the larger the corresponding area. Moreover, the corresponding time was distinguished by the cluster’s color, with cooler colors signifying earlier years and warmer colors indicating more recent ones.

In total, 14 clusters were found, and 13 clusters could be clearly presented. The details corresponding to each cluster are presented in Table 5, with the tag words highlighted in the cited subjects. The top 10 subjects are listed in order of the keyword count. The keywords contained in each cluster may be duplicated, and the duplication is reflected in the overlap area of the cluster mapping. The top-ranked subject by counts was “qualitative content analysis” (year: 2010) in Cluster 0 at 39, followed by “emotion regulation” (year: 2011) in Cluster 1 at 34, “oncology” (year: 2008) in Cluster 2 with a count of 33, and “grounded theory” (year: 2012) in Cluster 3 with a count of 33.

**Table 5:** The largest 13 clusters of painting therapy document co-citation, identified by subject headings.

Cluster ID	Size	Silhouette	Mean (year)	Top terms (LLR)
0	39	0.904	2010	Qualitative content analysis (9.14, 0.005); evaluation (4.55, 0.05); amplifications (4.55, 0.05); relaxation (4.55, 0.05); social change (4.55, 0.05); yoga (4.55, 0.05); eating disorder (4.55, 0.05); mind-body therapy (4.55, 0.05); incest survivors (4.55, 0.05); adults (4.55, 0.05); eurythmy (4.55, 0.05)
1	34	0.874	2016	Emotion regulation (8.38, 0.005); compassion (4.17, 0.05); spirituality (4.17, 0.05); symptoms (4.17, 0.05); compassion focused therapy (4.17, 0.05); maternal representation (4.17, 0.05); joint drawing (4.17, 0.05); art-based evaluation (4.17, 0.05); internal representations (4.17, 0.05); assessment (4.17, 0.05); bias (4.17, 0.05)
2	33	0.994	2008	Oncology (6.64, 0.01); arts-focused intervention (6.64, 0.01); museum object (6.64, 0.01); heritage-focused intervention (6.64, 0.01); health services research (6.64, 0.01); psychosis (6.64, 0.01); psychosocial (6.64, 0.01); occupational therapy (6.64, 0.01); art (6.07, 0.05); well-being (3.94, 0.05); rehabilitation (3.94, 0.05)
3	33	0.904	2012	Grounded theory (7.59, 0.01); therapeutic process (7.59, 0.01); adult mental health (4.1, 0.05); clinical practice (4.1, 0.05); survey (4.1, 0.05); depression (4.1, 0.05); transformation in parent–child relations (3.78, 0.1); counselling psychology (3.78, 0.1); major depression (3.78, 0.1); trauma (3.78, 0.1); medical art therapy (3.78, 0.1)
4	31	0.893	2015	Dementia (10.89, 0.001); visual art (10.09, 0.005); cognition (10.09, 0.005); art (7.39, 0.01); neurodegeneration (5.01, 0.05); acceptability (5.01, 0.05); music (5.01, 0.05); dance (5.01, 0.05); neuropsychology (5.01, 0.05); non-pharmacological intervention (5.01, 0.05); longitudinal (5.01, 0.05);
5	30	0.902	2016	Military (9.83, 0.005); traumatic brain injury (9.83, 0.005); veterans (9.83, 0.005); art therapy (7.97, 0.005); self-awareness (4.89, 0.05); trauma therapy (4.89, 0.05); suicide survivors (4.89, 0.05); avoidance (4.89, 0.05); bereavement (4.89, 0.05); military trauma (4.89, 0.05); drawings (4.89, 0.05)
6	23	0.953	2014	Pictures as signs of a neuroception of safety (6.37, 0.05); pictorial semiotics (6.37, 0.05); creating alongside (6.37, 0.05); art-based research (6.37, 0.05); assessment tool (6.37, 0.05); art-therapy (6.37, 0.05); art therapy and trauma (6.37, 0.05); trauma-therapy (6.37, 0.05); signs of dissociation and recovery (6.37, 0.05); altered state of consciousness and trauma (6.37, 0.05); increased positive affect and states (6.37, 0.05)
7	21	0.896	2017	Virtual reality (18.07, 1.0E-4); arts-based research (8.94, 0.005); psychomotor therapy (8.94, 0.005); psychotherapy (5.34, 0.05); poverty (4.45, 0.05); digital art therapy (4.45, 0.05); autonomic nervous system (4.45, 0.05); skin conductance (4.45, 0.05); school-based art therapy (4.45, 0.05); vr therapeutic setting (4.45, 0.05); anxiety (4.45, 0.05)
8	18	0.984	2018	Creative arts therapies (8.64, 0.005); nursing practice (6.13, 0.05); corona covid-19 (6.13, 0.05); tele-creative arts therapies (6.13, 0.05); tele-psychotherapy (6.13, 0.05); narrative gerontology (6.13, 0.05); tele-arts therapy (6.13, 0.05); nursing research (6.13, 0.05); photocollage (6.13, 0.05); creative self-efficacy (6.13, 0.05); older adults (6.13, 0.05)
9	15	0.946	2017	Cancer (12.22, 0.001); pediatric (10.98, 0.001); creative arts therapy (10.98, 0.001); quality of life (7.29, 0.01); cortisol (5.45, 0.05); il-6 (5.45, 0.05); posture (5.45, 0.05); psychosocial well-being (5.45, 0.05); complementary alternative therapy (5.45, 0.05); coloring (5.45, 0.05); survivorship care (5.45, 0.05)
10	13	1	2018	Intersectionality (11.52, 0.001); ghostly matters (5.71, 0.05); white supremacy (5.71, 0.05); resilience (5.71, 0.05); white antiracism (5.71, 0.05); museum (5.71, 0.05); intergenerational relations (5.71, 0.05); loneliness (5.71, 0.05); creative process (5.71, 0.05); self-reflexivity (5.71, 0.05); radical care (5.71, 0.05)
11	13	0.999	2017	e-mental health (7.15, 0.01); videoconferencing psychotherapy (7.15, 0.01); expressive arts therapy (7.15, 0.01); psychotherapists (7.15, 0.01); telemental health (7.15, 0.01); psychological wellbeing (7.15, 0.01); cognitive function (7.15, 0.01); telehealth (7.15, 0.01); mild cognitive impairment (7.15, 0.01); covid-19 (4.43, 0.05); psychotherapy (3.44, 0.1)
12	12	0.961	2017	Psychiatry (7.35, 0.01); manual (7.35, 0.01); expectancy (7.35, 0.01); patient preferences (7.35, 0.01); practice (7.35, 0.01); group arts therapies (7.35, 0.01); group (7.35, 0.01); intervention development (4.63, 0.05); arts therapies (3.63, 0.1); mental health (2.54, 0.5); art therapy (1.11, 0.5)
13	12	0.896	2012	Joint attention (7.15, 0.01); relational processes (7.15, 0.01); work-related stress (7.15, 0.01); care home (7.15, 0.01); older people (7.15, 0.01); staff groups (7.15, 0.01); arts for health (7.15, 0.01); art-viewing (7.15, 0.01); community (4.43, 0.05); art-making (4.43, 0.05); dementia (2.81, 0.1)

## 4. Discussion

### 4.1. Global trends in painting therapy research

This study performed a bibliometric analysis of painting therapy studies over the past decade. The findings reflected that painting therapy research has been held worldwide and provided scholars with suggestions for further research. In terms of the overall analysis of published articles, the characteristics, and countries or regions are summarized below.

First, the number of published articles per year has increased in the last decade. From 2011 to 2022, the annual volume of publications in painting therapy was divided into the following three phases: the beginning, second and third phases. The beginning phase was from 2011 to 2016 with <60 publications annually. The second phase was 2017–2019, which showed a steady increase in publication volume. The third phase was 2019–2022, with 2020 being a significant turning point as it was the first time that more than 100 articles were published. Overall, research on painting therapy received increasing attention from scholars between 2011 and 2022.

Second, the articles covered 61 countries or regions, and The United States had the maximum publication outputs, followed by Europe. According to the amount of citations on WoS, the citation counts of each study, and the H-index, publications on painting therapy from developed Western countries (e.g., the United States and the United Kingdom) had a greater impact than those from other countries or regions. Furthermore, China, as a leading example of developing countries, published 47 manuscripts, and was ranked the sixth in the productive countries.

Third, based on the data for the authors’ total amount of citations, publications counts, and the top 10 authors cited, the amount of publications for a certain author was generally low, and even the research by the author with the highest count of studies (Levwiesel R) accounted for only 1.15% of publications, suggesting a lack of leading scholars to conduct intensive and systematic research in this field.

### 4.2. Research focus on the research frontier and hot topics

Painting therapy had a range of research objects, and focused on special groups in recent years. The keyword co-occurrence mapping and keyword frequency and centrality indicated that the objects of painting therapy research were mainly women, adolescents, and children. The research encompassed a wide range of psychological problems, psychological disease groups, and physical disease groups. Research focused on individuals with psychological problems and diseases included groups of psychiatric diseases such as eating disorders, substance abuse, personality disorders, and posttraumatic stress disorder, in addition to depression (MacIntosh, 2017; Qiu et al., 2017; Hirano and Hosaka, 2018). For physical diseases, Cluster 2 (oncology) explored the application of painting therapy in oncology, with painting therapy mainly applied with patients with breast cancer (Czamanski-Cohen et al., 2019; Thyme et al., 2022), gynecological cancers (Wiswell et al., 2019), and other tumors (Puetz et al., 2013; Bozcuk et al., 2017; Elimimian et al., 2020). Painting therapy was also used with patients with Parkinson’s disease (Schofield, 2019), diabetes (Zamanifard et al., 2022), and children with gastrointestinal disorders (Kai et al., 2022). Alzheimer’s disease (Pongan et al., 2017) was the main focus of applicable diseases, with the highest keyword word frequency centrality; this also formed a cluster (Cluster #4) as the object of study on painting therapy. These results reflected the focus of painting therapy research on psychosomatic disorders. Studies also increasingly paid attention to special groups, including prisoners (Qiu et al., 2017; Gonzalez Barajas and Ho, 2020), pregnant women (Wahlbeck et al., 2020), children with autism (Durrani, 2019), people with low education and literacy levels (Crombie et al., 2022), and older people (Beauchet et al., 2020), reflecting medical humanism and community care.

In terms of research interventions, painting therapy can be applied alone, or combined with other therapies such as music therapy (Keidar et al., 2021), dance therapy (Kennedy et al., 2014), narrative therapy (Kruger and Swanepoel, 2017), mindful training (Jang et al., 2016), and cognitive behavioral therapy (Iosa et al., 2021), as well as other non-pharmacological treatment modalities. For patients with mental or physical illness, most interventions were pharmacotherapy combined with painting therapy with a view to exploring the effects of painting therapy in a safe and ethical manner. There were also studies (Feniger-Schaal et al., 2022) that explored the possibility of online/remote painting therapy, using virtual reality drawing to explore changes in patients’ mental impairment, physical functioning, and cognitive abilities (Iosa et al., 2021), which offered an innovative form of painting therapy intervention.

In terms of the outcome indicators, the efficacy of painting therapy was evaluated by most researchers using psychological scales for pre- and post-intervention efficacy assessment, and few studies applied physiological indicators as an efficacy assessment indicator. Because of the lack of reliability and validity of efficacy assessment tools based on painting elements (e.g., line, composition, color, prototype imagery), few studies used painting elements for efficacy assessment. Most studies focused on the changes in mental health indicators, and the cluster analysis also suggested that the frontier research hotspots included key words such as medical humanities, indicating that the evaluation of the painting therapy effectiveness covered changes in psychological scale indicators and the improvement of psychological conditions, as well as humanistic care for community rehabilitation, special populations, and clinical patients. For example, attention was paid to the improvement of patients’ quality of life (Bozcuk et al., 2017), reduction of pain (Stinley et al., 2015), improvement of interpersonal relationships (Durrani, 2019), improvement of psychological resilience (Huss et al., 2012), and increased self-esteem, self-efficacy, and happiness (Beauregard, 2014). A study (Versluis et al., 2022) that investigated the hair loss experience and feelings of patients with cancer receiving chemotherapy based on negative emotions caused by hair loss through painting therapy suggested that medical practitioners should pay attention to and take measures against hair loss in these patients.

It is noteworthy that the term “COVID-19” was found in the top 15 keywords with the strongest citation bursts, suggesting that painting therapy can also be applied to the psychological problems and diseases associated with the recent pandemic. In addition, with the development of modern technology, painting therapy is not limited to drawing on paper, but can be produced online using electronic applications and virtual drawing. This keyword may also relate to the dramatic rise in psychological problems during the COVID-19 pandemic and the increased difficulty of language-based and venue-required counseling. In this analysis, cluster 11 (e-mental health) was identified, which may offer an important direction for further exploring painting therapy.

What’s more, it is practical to conduct research on painting therapy in hospitals and colleges. With the shift from traditional medical systems to biopsychosocial medical networks, the role of psychological factors in the etiology and prognosis of physical illnesses has received more attention. On the one hand, physical illnesses can lead to psychological problems and even psychological disorders. For example, the diagnosis and treatment of cancer is often accompanied by changes in physical status and function, unpleasant side effects, reduced quality of life and impaired social relationships (Caruso et al., 2017); the prevalence of psychiatric disorders is much higher in cancer patients, with depression and anxiety being the two most common psychiatric symptoms (Niedzwiedz et al., 2019; Pilevarzadeh et al., 2019; Hashemi et al., 2020). On the other hand, mental health status is crucial to patient recovery, as it is associated with reduced quality of life, impaired social relationships, prolonged recovery time, poor treatment compliance and abnormal behaviour, as well as potentially shorter survival (Riba and Grassi, 2012). Painting therapy has relevance in hospital clinical practice as an affordable, easy-to-use and highly participatory psychotherapeutic tool that can improve patients’ psychological well-being and compliance. In addition, the dramatic increase in mental health problems among university students under the COVID-19 pandemic has exerted enormous pressure on the school counselling system (Charles et al., 2021; Mauer et al., 2022). Financial constraints and staff shortages have made face-to-face case consultations no longer an urgent solution for the currently large group of university students with psychological conditions, while painting therapy, an affordable, accessible and rapid assessment and treatment, may address this problem. Therefore, it is important to explore painting therapy in colleges and hospitals for practical reasons. Further exploration of the neuroendocrine mechanisms of painting therapy’s effect on mental health in the future may help to clarify the mechanisms of painting therapy and its scope of application.

### 4.3. Strengths and limitations

This study analyzed large-scale data from painting therapy publications over the past 10 years using CiteSpace. More comprehensive results, instead of simply reviewing articles and studies can be detected by CiteSpace. What’s more, bibliometric, as an in-depth analysis approach, enabled us to detect emerging trends and collaborations among authors and countries or regions.

This bibliometric study had some limitations. First, we only analyzed the publications in WoS; further reviews could select the articles from other canonical databases such as Scopus and PubMed. Second, the time constraints of the search might have led to bias in reference frequency. For instance, some studies were published recently, and they might not yet be cited as frequently as older articles. The search was conducted in July 2022, so full year data for 2022 cannot be presented. Third, quantitative analysis methods were used, and only limited qualitative analysis was conducted. Furthermore, CiteSpace was used to bibliometricly analyzed the publications, but the full count and fractional count system cannot be performed in this software to complicate the information. Therefore, further studies could use both quantitative and qualitative metrics to analyze the development of painting therapy in particular journals, and explore the relationship between citation rates, themes, and publications.

## 5. Conclusion

This analysis provides information of publications on trends in the painting therapy area from 2011 to 2022. First, it suggests several theoretical implications regarding publications to enable researchers to further promote their research. The annual publication volume of painting therapy research increased significantly over the past decade, with the overall trend in publication volume nearly tripling from 54 in 2011 to 154 in 2021. In 2022, however, as of July 2022, publication rate seems to have decreased, which needs to be followed up further. This study also enhances the holistic understanding of the global research structure in this field. A considerable degree of collaboration among different countries or regions and authors in painting therapy research was observed, which may expand the knowledge of painting therapy.

Second, this study has several other practical implications. Among the keywords citation bursts, the keywords “depression,” “women,” and “recovery” highlight the areas where painting therapy may be helpful in alleviating depression, benefiting women’s groups, and facilitating rehabilitation. The development of painting therapy is promising, and further studies could focus on the clinical impact of painting therapy for human psychosomatic health.

Finally, several studies demonstrated the effectiveness of painting therapy in improving physical and mental health. Furthermore, painting therapy can provide benefit to a range of individuals, especially women, adolescents, and patients with Alzheimer’s disease. It also has significant effects on certain special groups, such as prisoners, pregnant women, people with low education and literacy levels, or people with certain physical diseases such as cancer.

Meanwhile, there are some limitations to this article, such as the omission of certain publications led by the single database source and the specific search settings, a bias in reference frequency resulted from the time constraints of the search, information complication because of the inability of CiteSpace to perform full and fractional count systems. Therefore, further research in the future may consider additional databases and more loosely defined terms for data collection, perform quantitative exploration of the relationship between citation rates, themes, and publications, use both quantitative and qualitative indicators to analyze the development of painting therapies in specific journals, or employ with more advanced methods for comprehensive analysis, such as machine learning, text mining, and other advanced approaches. Mechanisms and criteria for assessing the efficacy of painting therapy are necessary for further research to advance the field, and researchers could develop painting therapy criteria to measure clinical practice.

## Author contributions

QL, HQ, and HS designed the paper. JY, YL, and JD edited the English text of a draft of this manuscript. QL and HQ revised the paper. HS and QL supervised the project. QL, JY, YL, JD, HS, and HQ participated in drafting and reviewing. All authors contributed to the article and approved the submitted version.

## Funding

This work is supported by Research Elite Funds for Institute of Analytical Psychology, Faculty of Humanities and Social Sciences, City University of Macau. (CUM: RE202201).

## Conflict of interest

The authors declare that the research was conducted in the absence of any commercial or financial relationships that could be construed as a potential conflict of interest.

## Publisher’s note

All claims expressed in this article are solely those of the authors and do not necessarily represent those of their affiliated organizations, or those of the publisher, the editors and the reviewers. Any product that may be evaluated in this article, or claim that may be made by its manufacturer, is not guaranteed or endorsed by the publisher.
